# Supplementary material for: Acetylation of H3K115 is associated with fragile nucleosomes at CpG island promoters and active regulatory sites
Source: eLife. 2026 Mar 4;14:RP108802. doi: 10.7554/eLife.108802 (PMC12959880; doi:10.7554/eLife.108802)
Supplement: Figure 4—source data 1. — LI-COR collection data shown below. [file elife-108802-fig4-data1.zip › Figure_4_Source_Data_1.pdf]

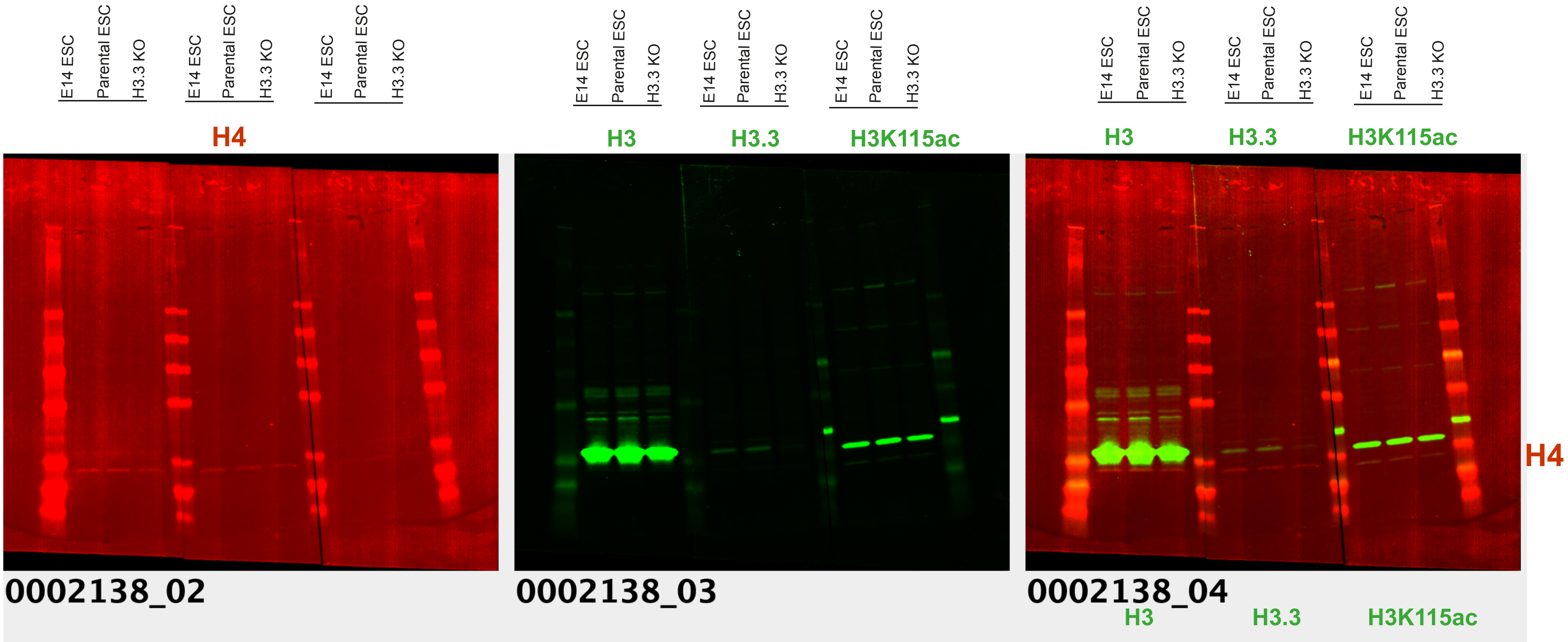

Images Table - All Images

#	Image ID	Acquire Time	Channels	Resolution	Intensities	Image Name	Comment
1	0002138_02	31 Jul 2024, 13:10:21	700, 800, RGB Epi	100 μm		0002138	
2	0002138_03	31 Jul 2024, 13:10:21	700, 800, RGB Epi	100 μm		0002138	
3	0002138_04	31 Jul 2024, 13:10:21	700, 800, RGB Epi	100 μm		0002138	

Image Display Values

Image ID	Channel	Color (Hex Code)	Minimum	Maximum	K	Opacity	Saturation	Blend
0002138_02	700	Red (#ff0000)	0.000762	30.6	0	1	N/A	N/A
0002138_03	800	Green (#00ff00)	0.0973	4.26	0	1	N/A	N/A
0002138_04	700	Red (#ff0000)	5.34	32.9	0	1	N/A	N/A
0002138_04	800	Green (#00ff00)	0.184	2.63	0	1	N/A	N/A
