# Supplementary figures and images for: Acetylation of H3K115 is associated with fragile nucleosomes at CpG island promoters and active regulatory sites
Source: eLife. 2026 Mar 4;14:RP108802. doi: 10.7554/eLife.108802 (PMC12959880; doi:10.7554/eLife.108802)

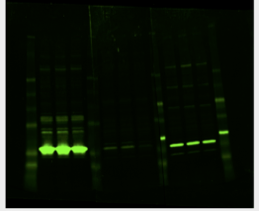

Supplement: Figure 4—source data 2. [file elife-108802-fig4-data2.zip › H3.tiff]

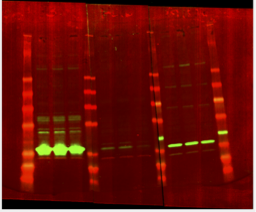

Supplement: Figure 4—source data 2. [file elife-108802-fig4-data2.zip › H3&H4.tiff]

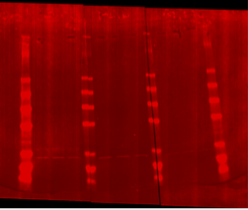

Supplement: Figure 4—source data 2. [file elife-108802-fig4-data2.zip › H4.tiff]

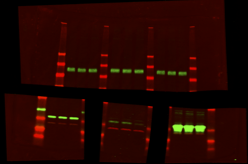

Supplement: Figure 4—figure supplement 1—source data 2. [file elife-108802-fig4-figsupp1-data2.zip › H3&H4.tiff]

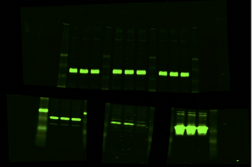

Supplement: Figure 4—figure supplement 1—source data 2. [file elife-108802-fig4-figsupp1-data2.zip › H3&tubulin.tiff]

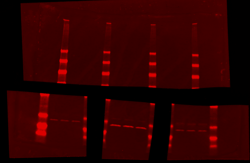

Supplement: Figure 4—figure supplement 1—source data 2. [file elife-108802-fig4-figsupp1-data2.zip › H4.tiff]
