# Supplementary material for: Acetylation of H3K115 is associated with fragile nucleosomes at CpG island promoters and active regulatory sites
Source: eLife. 2026 Mar 4;14:RP108802. doi: 10.7554/eLife.108802 (PMC12959880; doi:10.7554/eLife.108802)
Supplement: Figure 4—figure supplement 1—source data 1. — The gel was cut horizontally and the top half probed with antibody detecting β-tubulin as a loading control. The lower half has probed for H3/H4. LI-COR collection data shown below. [file elife-108802-fig4-figsupp1-data1.zip › Figure_4_figureSupplement_Source_data_1.pdf]

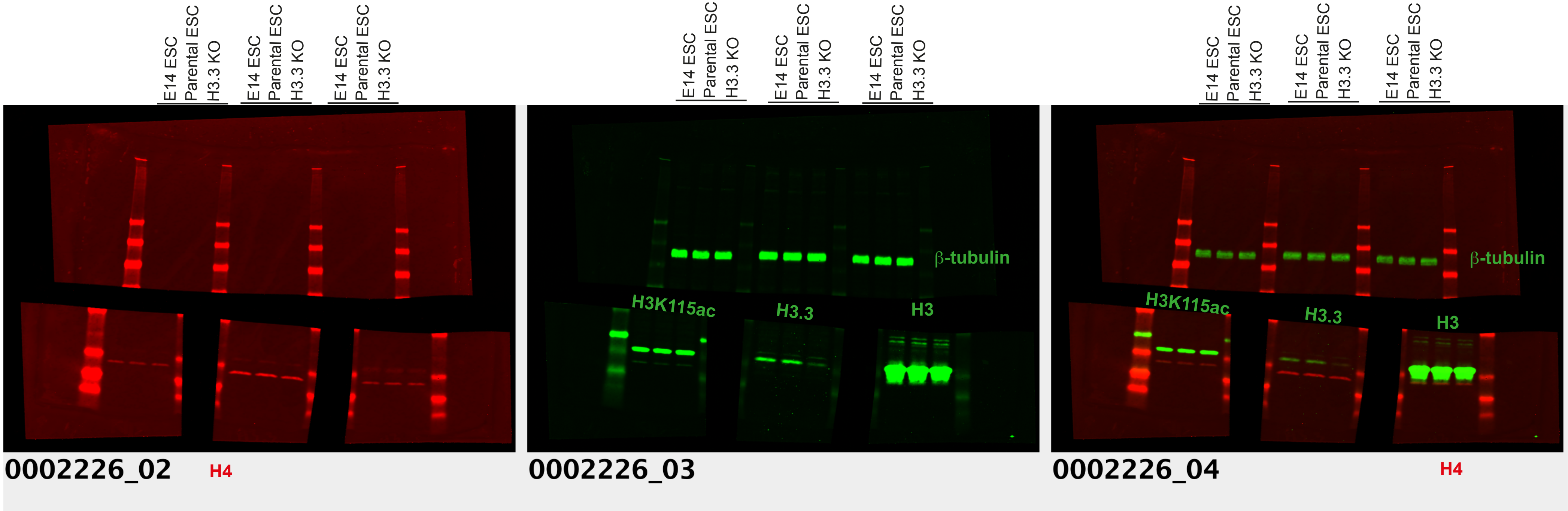

Images Table - All Images

#	Image ID	Acquire Time	Channels	Resolution	Intensities	Image Name	Comment	Image Modifications
1	0002226_02	8 Aug 2024, 13:44:25	700, 800, RGB Epi	100 μm		0002226		Rotated 180 Image ID: 0002226_01
2	0002226_03	8 Aug 2024, 13:44:25	700, 800, RGB Epi	100 μm		0002226		Rotated 180 Image ID: 0002226_01
3	0002226_04	8 Aug 2024, 13:44:25	700, 800, RGB Epi	100 μm		0002226		Rotated 180 Image ID: 0002226_01

Image Display Values

Image ID	Channel	Color (Hex Code)	Minimum	Maximum	K	Opacity	Saturation	Blend
0002226_02	700	Red (#ff0000)	0.00389	21.8	0	1	N/A	N/A
0002226_03	800	Green (#00ff00)	0.00330	2.15	0	1	N/A	N/A
0002226_04	700	Red (#ff0000)	0.00626	31.5	0	1	N/A	N/A
0002226_04	800	Green (#00ff00)	0.00946	6.02	0	1	N/A	N/A
